# Development of a randomised contrast detail digital phantom for observer detectability study

**DOI:** 10.2349/biij.2.3.e38

**Published:** 2006-07-01

**Authors:** MS Nizam, KH Ng, BJJ Abdullah

**Affiliations:** Department of Biomedical Imaging, Faculty of Medicine, University of Malaya, Kuala Lumpur, Malaysia

**Keywords:** Image quality, contrast detail, perception, receiver operating characteristics

## Abstract

The accuracy and the efficacy of radiological diagnosis depend, to a large extent, on the conditions under which radiographs and images are viewed. This mainly involves the luminance of the display devices and the ambient room illumination. We report a perceptual study to investigate the relationship between detectability and monitor luminance as well as ambient illuminance. A statistical test pattern was used in this study, and the test pattern was developed using Microsoft® Visual Basic 6. The test pattern contained a set of randomised contrast detail objects, that is, disks of different diameters (0.7, 1.0, 1.4, and 2.0 mm) and contrasts against a black background (2.7, 3.9, 5.5, and 7.8%), simulating lesions in digital images. The receiver operating characteristic (ROC) analysis was used in this study. The results indicated that a set of optimal viewing conditions exists and that it has a significant effect on detectability performance.

## INTRODUCTION

The accuracy and the efficacy of radiological diagnosis greatly depend on the conditions under which radiographs and images are viewed. This mainly involves the luminance of the display devices and the ambient room illumination [1-5]. A number of computer programs have also been used to evaluate image quality on softcopy display [6-8]. In this study, a randomised contrast detail digital phantom was developed to study the effect of CRT (cathode ray tube) display luminance and ambient illuminance on the perception of the observer. The digital phantom was so designed that the location of the pathology simulators within the phantom changed for every new use to ensure objectivity.

The digital phantom is available for download at http://www.biij.org/2006/3/e38/e38.exe.

## MATERIALS AND METHODS

The digital phantom was developed using Microsoft® Visual Basic 6 to generate 16 pathology simulators in the form of disks of different diameters (0.7, 1.0, 1.4 and 2.0 mm) and contrasts against a black background (2.7, 3.9, 5.5 and 7.8%) on CRT display monitor. These values were calculated with multiplicative factor of $\sqrt{2}$ according to *Cd = * constant, where *C*is the contrast of the test element with the background and *d* is the diameter of test element [9,10].

**Figure 1:**
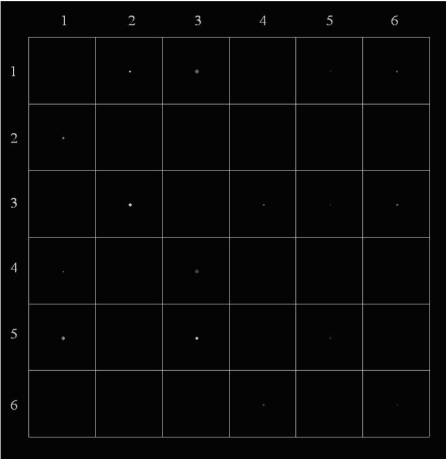
An image produced by the digital phantom showing the matrices (squares) containing randomly generated disks.

**Figure 2:**
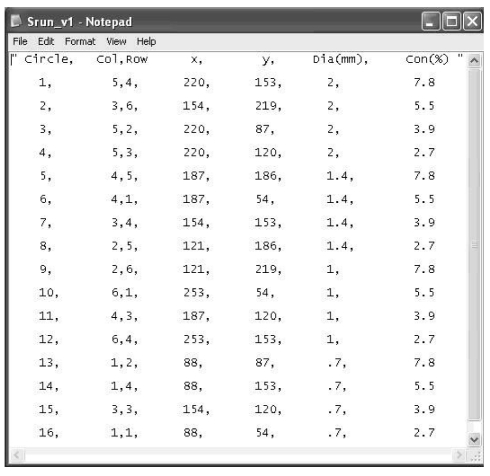
Text document produced by the digital phantom giving information on disk location (column and row, and x- and y- position), size, and contrast.

The digital phantom consisted of 6x6 squares (a total of 36 squares) at the centre of a 1280x1024 resolution CRT display monitor. The squares were labeled according to column and row from 1 to 6. Lines of the squares were 20% brighter than the black background. The 16 disks were generated randomly, and one disk could occupy only one square (Figure 1). A text document file with information about disk location, size, and contrast was also generated in the computer’s hard disk drive (C:\) each time the software was run (Figure 2).

Ten observers, who were radiographers with minimum three years of working experience, were asked to detect the disks from images created by the software. They were asked to use a 5-point scale, from 1 – definitely absent to 5 – definitely present. Their answers were compared with the correct answers provided by the text document file.

Display luminance measurements were taken according to the method recommended by Parsons *et al.* [11] using a luminance level meter (Mavo-Monitor, Gossen-Metrawatt Gmbh, Nürnberg, Germany). The contrast level was set to 100%. After 30 minutes of warm up, the 100% grey-level square of a SMPTE test pattern was zoomed so that it filled the display area. The screen was divided into nine squares and measurements of the luminance output were taken at the centre of each of these squares. The luminance was adjusted to the required level using the brightness control.

Ambient illuminance measurements were taken according to the method recommended by Moores *et al.* [12] using a photometer (model PMLX, Quantum Instruments Inc., New York, USA). All types of display were switched off, and the photometer was used to measure the level of room illumination at a point 30 cm from the display. The room illuminance level was adjusted using a light dimmer.

The phantom was tested on a 21-inch colour CRT monitor (Sony model GDM-500PS, Sony Electronics Inc., California, USA) with 1280x1024 resolution and 32 bit at 85 Hz refresh rate. The CRT monitor luminance was 120.0±0.9 and 80.0±0.9 cd/m^2^, respectively, each viewed under 30.0±0.1, 50.0±0.1, and 70.0±0.1 lux room illumination. Observers were given approximately 10 minutes for their eyes to adapt to the room illumination before commencing to evaluate each test image.

## RESULTS

**Figure 3:**
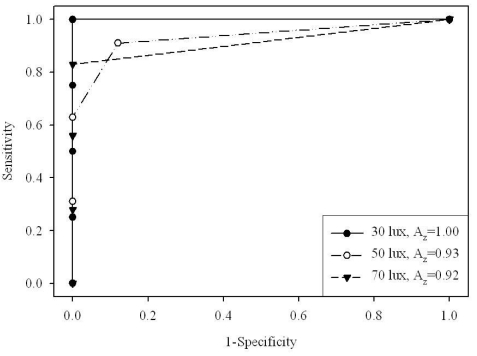
ROC curve (*n*=10) for 1.0 mm disks on 120 cdm^-2^ CRT monitor.

**Figure 4:**
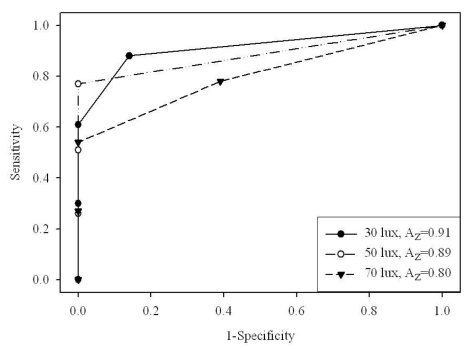
ROC curve (*n*=10) for 1.0 mm disks on 80 cdm^-2^ CRT monitor.

Overall ROC curves and area under the curve values (A_z_) were perfect or almost perfect for big size disks (2.0 and 1.4 mm), but as the disk size decreased observer performance also decreased. Higher ambient illumination decreased observer performance, and its effect was more pronounced with lower display luminance (80 cd/m^2^) compared with higher display luminance (120 cd/m^2^). Figures 3 and 4 show the ROC curves and A_z_ values obtained for 1.0 mm disks on 80 and 120 cd/m^2^ CRT monitors, respectively.

## DISCUSSION AND CONCLUSION

The result of this study concerning the effect of display luminance and ambient illuminance is in agreement with previous findings [1-5]. Based on the result, a high display luminance with minimum reasonable ambient illuminance is recommended to optimise softcopy reporting. Other relevant factors that may influence the perception tasks involved in the radiology reading room session have been listed by Wang and Langer [13]. They are: (1) spatial and contrast resolution of the display device; (2) brightness and the displayed luminance range of the monitor (or film/viewbox); (3) uniformity of the display system luminance; (4) extraneous light in the reading room (such as bright, unmasked areas on the monitor and light reflected off the monitors); (5) displayed field size (field of view); (6) viewed object orientation; (7) image motion and flickering of display device; (8) signal to noise ratio of the displayed image; (9) magnification and zooming functions; and (10) user interface of the workstation.

The performance of softcopy reporting has become an important issue due to the rapid introduction of digital radiology in hospitals. This randomised contrast detail digital phantom is another valuable tool for observer detectability study and quality control of softcopy display. It could be used to evaluate the new generation of flat screen displays being used increasingly in radiology departments.

## References

[1] Roehrig H, Krupinski E (1998). Image Quality of CRT displays and the effect of brightness of diagnosis of mammograms. J Digit Imaging.

[2] Flynn MJ, Badano A (1999). Image quality degradation by light scattering in display devices. J Digit Imaging.

[3] Hemminger BM, Dillon AW, Johnston RE (1999). Effect of display luminance on the feature detection rates of masses in mammograms. Med Phys.

[4] Roehrig H, Krupinski EA, Furukawa T (2001). Evaluation of a flat CRT monitor for use in radiology. J Digit Imaging.

[5] Chakrabarti K, Kaczmarek RV, Thomas JA (2003). Effect of room illuminance on monitor black level luminance and monitor calibration. J Digit Imaging.

[6] Wang J, Peng Q (2002). An interactive method of assessing the characteristics of softcopy display using observer performance tests. J Digit Imaging.

[7] Davydenko G, Gurvich V, Smekhov M (2002). Application of StatPhantom software for image quality evaluation. J Digit Imaging.

[8] Norrman E, Gardestig M, Persliden J (2005). A clinical evaluation of the image quality computer program, CoCIQ. J Digit Imaging.

[9] Rose A (1973). Vision: Human and Electronic.

[10] Burgess AE (1999). The Rose model, revisited. J Opt Soc Am A Opt Image Sci Vis.

[11] Parsons DM, Kim Y, Haynor DR (1995). Quality control of cathode-ray tube monitors for medical imaging using a simple photometer. J Digit Imaging.

[12] Moores BM, Watkinson SA, Henshaw ET, Pearcy BJ (1991). Practical Guide to Quality Assurance in Medical Imaging.

[13] Wang J, Langer S (1997). A brief review of human perception factors in digital displays for picture archiving and communications systems. J Digit Imaging.

